# Proteogenomic Analysis of Breast Cancer Transcriptomic and Proteomic Data, Using *De Novo* Transcript Assembly: Genome-Wide Identification of Novel Peptides and Clinical Implications

**DOI:** 10.1016/j.mcpro.2022.100220

**Published:** 2022-02-26

**Authors:** P.S. Hari, Lavanya Balakrishnan, Chaithanya Kotyada, Arivusudar Everad John, Shivani Tiwary, Nameeta Shah, Ravi Sirdeshmukh

**Affiliations:** 1 Mazumdar Shaw Center for Translational Research, Narayana Health, Bangalore, India; 2 Simulation and Modeling Sciences, Pfizer Pharma GmBH, Berlin, Germany; 3 Institute of Bioinformatics, International Tech Park, Bangalore, India; 4 Health Sciences, Manipal Academy of Higher Education, Manipal, India

**Keywords:** proteogenomics, breast cancer, *de novo* transcript assembly, alternative splicing, CPTAC, ALDOA, aldolase A, BRCA, breast cancer, CPTAC, The Clinical Proteomic Tumor Analysis Consortium, CXCL16, C-X-C motif chemokine ligand 16, FADD, Fas-associated death domain, FGFR, fibroblast growth factor receptor, FLT1, Fms-related receptor tyrosine kinase 1, HER2, human epidermal growth factor receptor 2, iTRAQ, isobaric tag for relative and absolute quantitation, lncRNA, long noncoding RNA, MS, mass spectrometry, nORF, novel ORF, PLCB3, phospholipase C beta 3, PPP2R2A, protein phosphatase 2 regulatory subunit B alpha, PSM, peptide-spectrum match, RPA1, replication protein A1, sORF, small ORF, TCGA, The Cancer Genome Atlas

## Abstract

We have carried out proteogenomic analysis of the breast cancer transcriptomic and proteomic data, available at The Clinical Proteomic Tumor Analysis Consortium resource, to identify novel peptides arising from alternatively spliced events as well as other noncanonical expressions. We used a pipeline that consisted of *de novo* transcript assembly, six frame-translated custom database, and a combination of search engines to identify novel peptides. A portfolio of 4,387 novel peptide sequences initially identified was further screened through PepQuery validation tool (Clinical Proteomic Tumor Analysis Consortium), which yielded 1,558 novel peptides. We considered the dataset of 1,558 validated through PepQuery to understand their functional and clinical significance, leaving the rest to be further verified using other validation tools and approaches. The novel peptides mapped to the known gene sequences as well as to genomic regions yet undefined for translation, 580 novel peptides mapped to known protein-coding genes, 147 to non–protein-coding genes, and 831 belonged to novel translational sequences. The novel peptides belonging to protein-coding genes represented alternatively spliced events or 5′ or 3′ extensions, whereas others represented translation from pseudogenes, long noncoding RNAs, or novel peptides originating from uncharacterized protein-coding sequences—mostly from the intronic regions of known genes. Seventy-six of the 580 protein-coding genes were associated with cancer hallmark genes, which included key oncogenes, transcription factors, kinases, and cell surface receptors. Survival association analysis of the 76 novel peptide sequences revealed 10 of them to be significant, and we present a panel of six novel peptides, whose high expression was found to be strongly associated with poor survival of patients with human epidermal growth factor receptor 2–enriched subtype. Our analysis represents a landscape of novel peptides of different types that may be expressed in breast cancer tissues, whereas their presence in full-length functional proteins needs further investigations.

## Highlights

• Novel protein variants and peptides from noncoding sequences are rapidly emerging.
• Mining of mass spectrometry data using proteogenomic analysis reveals such entities.
• Novel peptides from coding and noncoding sequences identified in breast cancer.
• Novel peptides mapped to cancer hallmark genes in breast cancer.
• Panel of novel peptides with prognostic potential found for HER2-enriched subtype.

## Graphical abstract

**Figure:**
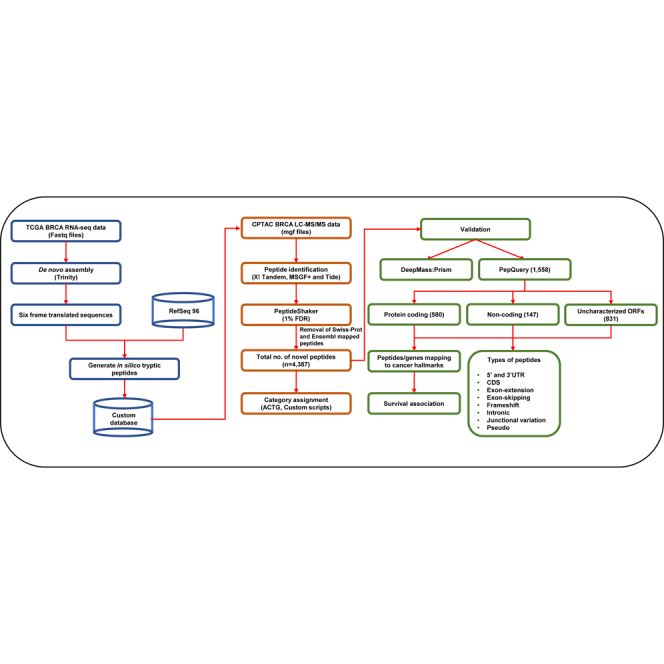

## In Brief

Alternative splicing of known protein-coding genes and expression of noncoding sequences of the human genome are increasingly expanding the functional diversity of proteins. These events may be specific to cell type or physiological condition of the cells and may be deregulated in cancer. Using proteogenomic analysis, we have identified novel peptides in breast cancer that arise from such events. High expression of some of them is associated with patients' survival and may be studied as prognostic indicators.

As per the recent database information, human genome is believed to have close to 20,000 protein-coding genes, about 2,000 of them are yet to have experimental evidence for their existence (1). Rest of the genome was until recently considered to be noncoding and harbors genes for several types of RNAs and other intergenic sequences that may have elements of long-range interactions involved in chromatin remodeling and regulation of gene expression. There has been growing evidence indicating that these gene sequences that were once thought to be noncoding may actually be translated (2, 3) and reveal peptides mapping to long noncoding RNAs (lncRNAs), pseudogenes, or other sequences. The classic examples of protein-coding pseudogenes include phosphoglycerate mutase 3, pseudogene (*PGAM3*), and *OCT4-pg1.* A small ORF (sORF) derived from the pseudogene, *BRAFP1*, has been reported to enhance tumorigenesis in thyroid tumors. Some of the peptides from sORFs within the lncRNA sequences, such as, SPAR, Minion, HOXB-AS3, and NOBODY, were shown to be associated with the progression of various cancers (4, 5, 6).

Proteogenomic approaches integrating RNA-Seq and mass spectrometry (MS) data allow identification of the translational potential of such sequences as well as mutations, alternate start sites, alternative splicing events, and gene fusions at the protein level. Many of them have been reported to have cell type–specific expression and are also associated with tumor development (7). Advances in next-generation RNA sequencing and high-resolution MS have greatly enhanced the application of proteogenomic methods especially in the field of cancer biology, to identify tumor-specific novel peptides, splice variants, and fusion products that can serve as diagnostic or prognostic biomarkers or as a source of tumor neoantigens with immunotherapy potential (8, 9). Considering this, The Human Proteome Project has also included this as a thematic objective. In one of the major initiatives, The Clinical Proteomic Tumor Analysis Consortium (CPTAC) has carried out proteogenomics analysis of a number of cancers, such as breast (10), colorectal (11), ovarian cancer (12), and glioblastoma providing new molecular insights such as protein-centric subtypes (13). In addition, proteogenomic characterization of hepatitis B-related hepatocellular carcinoma and early onset gastric cancer has been reported by other research groups (14, 15).

Proteogenomic analysis carries several challenges especially in terms of the use of *in silico*-developed custom databases for peptide searches, their size, and reliability of novel peptide identifications (16). Generation of sample-specific databases derived from RNA-Seq data for MS searches has been shown to be beneficial for identifying any sample-specific variability resulting from transcriptional and post-transcriptional processes (17, 18). Several tools and pipelines have been developed for customized database generation for performing proteogenomic analysis, including QUILTS (19), PGA (20), PGMiner (21), Splicify (22), ASV-ID (23), JUMPg (24), and ProteomeGenerator (25). Recently, an integrated proteogenomic analysis workflow was developed by Zhu *et al.* (26) for the discovery, curation, and validation of novel as well as single amino-acid variant peptides.

Unlike using the reference genome, *de novo* transcript assembly offers the advantage of detecting full transcript of a gene sequence and any variation therein such as alternatively splice events and relate them at protein level (27). Mittal *et al.* (28) reported the identification of a large number of novel fusion-gene transcripts by *de novo* assembly of breast cancer (BRCA) transcriptome. In our study, we developed a proteogenomics pipeline, which builds the custom database using longest ORF from six-frame translation of *de novo* assembled transcripts to identify novel peptides and protein variants. In a pilot study, using this pipeline, we reanalyzed publicly available data that include patient-specific RNA-Seq data for BRCA samples drawn from The Cancer Genome Atlas (TCGA) and the MS-based proteomic data for the same specimens generated by CPTAC. We identified translational evidence for several noncoding sequences as well as alternative splicing events of genes.

## Experimental Procedures

### Datasets

For the primary proteogenomics analysis, we used BRCA RNA-Seq and MS proteomics data for a matched sample cohort obtained from TCGA and CPTAC resources, respectively. A total of 105 BRCA samples were used for the analysis. TCGA RNA-Seq data generated for 105 BRCA samples using Illumina HiSeq sequencer to a depth of 60 to 150 million reads per sample was downloaded. The respective RNA-Seq fastq files were downloaded from GDC legacy archive (https://portal.gdc.cancer.gov/legacy-archive). Of 105 samples, there were 25 basal-like, 29 luminal A, 33 luminal B, and 18 human epidermal growth factor receptor 2 (HER2)-enriched tumors. The fastq files of BRCA samples were concatenated by merging right and left reads separately. Proteomics data generated for the same samples were downloaded from CPTAC data portal (https://cptac-data-portal.georgetown.edu/cptac/s/S029). Proteomic data consisted of a total of 36 isobaric tag for relative and absolute quantitation (iTRAQ) (4-plex) experiments, each with three tumor samples (labeled with 114, 115, and 116 iTRAQ labels) and one pooled internal reference (iTRAQ label 117). Twenty-four mzML files were obtained from each iTRAQ experiment, corresponding to 24 LC fractions of the protein digests analyzed on Orbitrap Q Exactive mass spectrometer (Thermo Fisher Scientific).

### *De Novo* Transcript Assembly and Development of Custom Database

*De novo* transcript assembly of the combined RNA-Seq fastq files for each set of three samples, used in 36 iTRAQ (4-plex) experiments, was carried out using Trinity (version 2.8.3) (developed by Broad Institute; https://github.com/trinityrnaseq/trinityrnaseq/wiki), after trimming low-quality reads through Trimmomatic option (27). Trinity fastq files obtained for each set of samples were then run through the function createProDB4DenovoRNA-Seq in PGA (version 1.16.0) to obtain six-frame translated protein sequences for the assembled transcripts. Only the longest protein sequences were selected and digested with trypsin, *in silico*, and database generated. Tryptic peptide sequences for proteins from Human National Center for Biotechnology Information RefSeq Release 96 (no. of NP entries = 54,216) were also incorporated into the database for identifying known peptides along with the novel ones, using the respective labels.

### Database Searching and Identification of Novel Peptides

mzML files of the MS data were converted to mgf files with PeakPicking MS2 spectra option using msConvert (3.0.9393) (29). The custom database generated for the sample sets used in each of the 36 experiments was searched against the respective mgf files using three search engines available in SearchGUI (version 3.3.15)—X!Tandem, MSGF+, and Tide (30). The decoy sequences were created by reversing the target sequences in SearchGUI. Carbamidomethylation on cysteine residues, iTRAQ reagent tags on lysines, and peptide N-termini were chosen as static modifications, whereas oxidation of methionine, acetylation of protein N-termini, and deamidation on asparagine were chosen as dynamic modifications. Precursor ion tolerance and fragment ion tolerance were set as 10 ppm and 0.05 Da, respectively. The number of missed cleavages allowed was 2, and the length of peptides allowed was between 7 and 30 amino acids (however, the average length of the peptides identified was 12 amino-acid residues; see Results section). PeptideShaker (version 1.16.40) (31) was used to combine the results of aforementioned three search engines and to convert the scores of search engines to posterior error probability values. The corresponding peptide-spectrum matches (PSMs) were marked as significant by a quality filter in PeptideShaker, which is based on mass deviation and fragment ion coverage. Peptide confidence was calculated based on the combined score of PSMs of the respective peptides. The significant peptide output data from PeptideShaker with 1% false discovery rate were taken for further processing. Peptides that mapped to multiple genomic locations were removed. We in addition mapped these peptides to ENSEMBL to verify and exclude any of the identified sequences that are reported there. Peptides that did not map to RefSeq and ENSEMBL protein IDs were considered as novel.

To add further strength to the identification of the novel peptides, we tried to crossverify the identifications using additional verification tools, namely PepQuery (Standalone version, 1.3.0) (developed by CPTAC Consortium; http://www.pepquery.org/) (32) and DeepMass:Prism (developed by Verily Life Sciences in collaboration with Cox Lab, Max Planck Institute of Biochemistry; https://github.com/verilylifesciences/deepmass/tree/main/prism) (33). PepQuery is a peptide-centric search engine developed by CPTAC and based on millions of experimental fragmentation spectra for peptide sequences detected in cancer proteomic datasets. PepQuery could be used for fast proteomic validation of novel peptides. Conceptually similar to BLAST, it allows querying the peptide MS/MS spectral database with a sequence of interest. Given a novel peptide sequence of interest, PepQuery comprehensively analyzes and searches for the PSMs in the input spectral database, sequentially filtering PSMs of known peptide sequences (RefSeq) and randomly reshuffled target peptide sequences and narrows down the most probable spectral matches for the target peptide sequences, which could now be scored as novel peptides, and the two spectra (PepQuery revealed and the original) may be compared. An additional advantage of using PepQuery is that it reduces the false positives by considering the sequence modifications as well. The PSM significance score is determined in terms of a permutation *p* value calculated based on randomly shuffled peptide sequence of interest. DeepMass:Prism is a prediction tool that uses neural network based deep/machine learning algorithm trained on a library of millions of experimental fragmentation spectra of unmodified tryptic peptides and can predict peptide fragment intensities, which are usually dependent upon fragment charge, the amino acid chemistry, and the sequence context. Although this tool has been trained for verification of data-dependent peptide identifications or as a guide for spectral prediction in data-independent acquisition, we have used it to predict the fragment intensities in of the fragment ions for the novel peptide sequences and using them for the verification of the experimental spectra that identified the peptide sequence.

While we briefly discuss the DeepMass:Prism validation output (see Results section), we used the PepQueryverified novel peptides for clinicobiological interpretations. Wherever possible, we also assessed the confidence of the novel peptide identification through additional comparisons with known datasets (see Results section). Furthermore, for the select, biologically, or clinically significant novel peptides, the quality of the MS/MS spectra was evaluated through manual inspection using PDV (version 1.5.1) (34). Spectra of individual peptides were extracted from their corresponding mgf files, and the exported spectra were loaded on to PDV, and the coverage of b and y ions within each peptide sequence was examined to ensure that the novel peptide identification conformed to good spectral quality as judged by number of assigned ions (80%), signal-to-noise ratio, and sequence contiguity.

### Novel Peptide Categorization

The identified peptides were processed to map the sequences to their corresponding genomic locations using the tool, ACTG (Standalone version 1.11) (developed byPaek Lab, Hanyang University, Seoul, Korea; http://prix.hanyang.ac.kr/ACTG/search.jsp) (35), and to classify them into various categories, namely—peptides mapping to known protein-coding genes, those mapping to known noncoding genes, and third, peptides mapping to hitherto uncharacterized potential novel ORFs (nORFs). Peptides belonging to the protein-coding genes were further classified into 5′ or 3′ UTRs, alternative splicing types such as, exon extension, exon skipping, frameshifts, junctional variations, and others representing novel coding frames within the known gene (coding DNA sequence). Similarly, peptides originating from nonprotein-coding transcripts were grouped into pseudogenes, lncRNAs, and others. We also recategorized exonextension peptides as exon overlapping or intronic peptides.

For the purpose of the aforementioned classification, custom scripts were developed that included Bedtools closest (36). Peptides that mapped to Swiss-Prot or multiple genomic locations were removed, and BlastP analysis of the remaining peptide sequences was performed to identify sequences that are “not an ORF.” All the novel peptides from 36 experiments were merged, and the total number of PSMs for each peptide was calculated.

### Biological and Clinical Relevance of Novel Peptides

Novel peptide identifications belonging to the known protein-coding genes were mapped to cancer hallmark-associated genes (http://bio-bigdata.hrbmu.edu.cn/CHG/nav_download.html) to assess their biological and clinical significance. Survival analysis of these candidates was carried out using TCGA patient data for BRCA (TCGA-BRCA). The novel peptides were mapped to the corresponding transcripts, and their expression levels determined (at RNA level from the TCGA resource) by using the entire RNA-Seq data of BRCA samples used, in various subtype-specific manner. Chromosomal locations of the peptide sequences obtained through ACTG were processed using bamstat04 (http://lindenb.github.io/jvarkit/BamStats04.html) across all bam files of TCGA-BRCA data. Average coverage obtained for each sequence in each sample was converted to counts per million values. Survival analysis was then performed using the Kaplan–Meier method within each subgroup of BRCA subtypes. We also checked for any influence on survival by the parent gene sequences by independently running the survival analysis of those sequences.

## Results

### Novel Peptide Identification Using *De Novo* Transcript Assembly–Based Proteogenomic Analysis

There are several pipelines reported in the literature that have been used for proteogenomic analysis to identify alternatively spliced variations. Some of the proteogenomic pipelines available in the literature and used by other investigators during the last 5 years are given in Table 1.

**Table 1:** List of proteogenomics tools/pipelines and their salient features

Name of pipeline/tool	Input data (source)	Custom database creation	Search engines	Splice-type interpretation	Differential expression information	Reference
IPAW	RNA-Seq, MS/MS raw data (A431 cells)	Genomic sequence database or transcript assembly aligned with genomic sequence. Six-frame translation of genomic sequences, no integrated transcript assembler	MSGF+	Present	Not present	(26)
JUMPg	RNA-Seq, MS/MS raw data (Alzheimer’s disease postmortem brain, multiple myeloma cell line [ANBL6])	Reference genomic sequence used for custom protein database through three-frame translation and six-frame translation *de novo* custom protein database creation	Single tag–based in-house built multistage search engine	Present	Not present	(24)
PGMiner	RNA or complementary DNA sequence, MS/MS raw data (*Toxoplasma gondii* RH strain)	User-defined custom database creation with three-frame translation with an option of six-frame translation based on reference genome sequence	MSGF+, OMSSA, and X!Tandem	Present	Not present	(21)
Splicify	RNA-Seq fastq files, MS/MS raw data (colorectal cancer cell line SW480)	Reference genome sequence based on three-frame translation and custom protein database creation	MaxQuant	Present	Present	(22)
ASV-ID	RNA-Seq fastq files, MS/MS raw data (human embryonic kidney 293, HepG2, HeLa, and MCF7 cell lines)	Reference genome sequence based on three-frame translation and custom protein database creation	Comet	Present	Present	(23)
PGA	vcf, bed, and gtf files from RNA-Seq, MS/MS raw data. (Jurkat cell line)	Reference genome sequence based on three-frame translation or six-frame translation of *de novo*-based transcript custom protein database creation	One default search engine	Present	Not present	(20)
ProteomeGenerator	RNA-Seq, MS/MS raw data (K052 leukemia cells)	Reference genomebased sequence alignment, Reference or *de novo*-based transcript assembly and custom protein database creation	MaxQuant	Present	Not present	(25)

PubMed literature was searched for the last 5 years using proteogenomics tools and pipelines as the keyword, and some of the major tools/pipelines were examined for the analytical details. The list given in the table is not exhaustive but represents major tools/pipelines used. The custom pipeline used in CPTAC analysis is not listed in this table but discussed under the Results section.

For customized protein database from transcriptomics data, the RNA-Seq reads are assembled into full-length transcripts, using one of the two general approaches: genome alignment and *de novo* transcript assembly. As shown in the table, most of the pipelines use transcript assembly using reference genome sequence. However, *de novo* transcript assembly methods have the greater capability of identifying novel transcripts that cannot be identified through reference genome-alignment methods either because of errors in the reference genome sequence or because of the absence of the novel sequence. Several tools such as Trinity (used by us), Trans-ABySS, SOAPdenovo-Trans, and SPAdes (37) are often employed. Yet they are not free of errors needing further evaluations. Although, merging the results obtained from multiple assembly tools followed by further evaluation may yield better results, we have used Trinity for transcript assembly for the development of custom protein database for searching MS/MS files. Long-read sequencing technologies have the potential to circumvent challenges of *de novo* transcriptome assembly. There are platforms such as PacBio and Nanopore technologies, with which up to 10 kb long reads are achieved; however, these platforms are yet to be under routine use.

In the studies reported in Table 1, either three-frame or six-frame translation *in silico* was used for creating the custom database. In JumpG, the two databases were used in tandem—first three-frame translated sequences for searching MS/MS files followed by six-frame database for residual data not mapping to three-frame data. JumpG has used in-house built search engine, whereas other pipelines have used publicly available single search engine or a combination of multiple engines. JumpG has also incorporated semitryptic peptides in their database creation (not shown in the table). One of the pipelines, PGA, also uses *de novo* transcript assembly for the development of custom protein database.

CPTAC has earlier carried out proteogenomic analysis with RNA-Seq data drawn from TCGA resource for 105 patients belonging to defined BRCA subtypes, and MS/MS data were generated by CPTAC for the same samples, through 36, 4-plex iTRAQ-based MS/MS experiments (see Experimental Procedures section). A custom pipeline was used for that study. The pipeline used subtype-specific six-frame translated custom database created through QUILTS pertaining to somatic and germline single nucleotide variants, RNA-Seq predicted splice junctions and fusion genes. Spectrum Mill was then used to search the proteomic data against the custom database to identify splice variants.

**Fig. 1:**
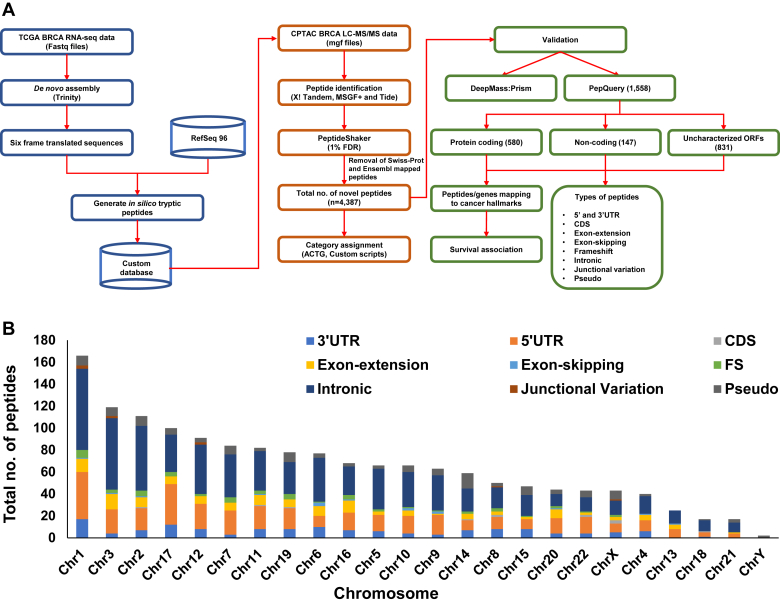
**Proteogenomic analysis and identification of novel peptides.***A*, a schematic view of the proteogenomic pipeline. Breast cancer transcriptomic and proteomic data from CPTAC resource was used for the analysis. The pipeline includes *de novo* assembly of RNA-Seq reads followed by six-frame translation for custom database creation to search against the MS/MS files from the proteomics analysis. The custom database generated for each of the samples was searched against the respective mgf files using the search engines, X!Tandem, MSGF+, and Tide. PeptideShaker was used for integrated identification of the candidate peptides and their corresponding proteins. The known peptides (RefSeq) were then filtered out from the total identifications to get the list of novel peptides, which were then subjected to ACTG analysis followed by categorization into different kinds of peptide categories using custom scripts as described under Experimental Procedures section. The novel peptides obtained were then validated using PepQuery. The novel peptides validated by PepQuery were categorized into those that map to protein-coding genes, noncoding genes, and uncharacterized ORFs. Numbers shown in *brackets* represent number of novel peptides in the respective groups. The different types of novel peptides obtained after ACTG categorization are also shown. The novel peptides mapping to known protein-coding genes were mapped to cancer hallmark genes and further assessed for clinical relevance in breast cancer by carrying out survival analysis. Validation with DeepMass:Prism is briefly discussed in the Results section. *B*, chromosome-wise distribution of the PepQuery-validated peptides as revealed by ACTG and their respective categories, indicated by the color code, is shown. The details of the categories are explained under Experimental Procedures section.

We have analyzed the same datasets downloaded from CPTAC resource, using our pipeline shown in Figure 1*A*. The main features of our pipeline are use of *de novo* transcript assembly of RNA-Seq data as discussed previously, six-frame translation of the assembled transcripts, and use of three integrated search engines. Major advantage of *de novo* transcript assembly is that it provides evidence for the longest transcript carrying the novel peptide sequences. *De novo* transcript assembly of RNA-Seq data was carried out using Trinity. Subtype-specific transcripts were then obtained to match with each of the sample groups used for 36 proteomic (4-plex iTRAQ) experiments. On an average 110,000 transcripts were identified from the combined fastq files for each group. Furthermore, RNA-Seq samples used were not strand specific; so we looked at all six frames. We used createProDB4DenovoRNA-Seq in PGA (version 1.16.0) to obtain six-frame translated protein sequences for the assembled transcripts, and only the longest protein sequences were used for *in silico* trypsin digestion for database generation. This yielded around 180,000 protein sequences from each set of samples. These sequences were then merged with the protein sequences from Human RefSeq, Release 96 to constitute a custom database to search against the MS/MS files from the respective experiment. MS/MS search using SearchGUI (see Experimental Procedures section) followed by processing with PeptideShaker resulted in the identification of all peptides mapping to RefSeq and the Trinity transcript sequences. The peptides that did not map to RefSeq and Swiss-Prot were considered as novel peptides. A total of 4,791 novel peptides were identified with 13,063 PSMs, about 30% being with multiple PSMs. We in addition mapped these peptides to ENSEMBL to examine the presence of any known sequences and found about 404 of them to be already reported and so filtered them out to take remaining 4,387 for further analysis (supplemental Table S1). We applied peptide length in the range 7 to 30 amino-acid residues for the database search; however, the average length of the novel peptides identified is about 12. Although identified using a combination of three search engines and stringent scoring criteria, these peptide identifications are still likely to have false positives and need to be subjected to rigorous further verifications by additional ways to enhance the accuracy of identifications. In our study, we used additional validation tools, cross-verification of the presence of novel peptides in other datasets, or even manual quality check and annotation of the MS/MS spectra wherever necessary and possible.

For this purpose, we first carried out verification of the 4,387 novel peptide identifications with PepQuery validation tool developed by CPTAC (37) and based on a large number of peptide fragmentation spectra reported in cancer proteomics datasets. The novel peptide sequences were re-searched against the MS/MS spectral data of the respective experiments from which they were originally identified, using PepQuery workflow (see the Experimental Procedures section). Carbamidomethylation of C, iTRAQ 4-plex of K, iTRAQ modifications (4-plex) at N-terminal region were used as fixed modifications, whereas oxidation of M, acetylation of protein N-terminal region, and deamidation of N were used as variable modifications. Fragment ion tolerance was set as 0.05 Da. Hyperscore was used for PSM scoring, and unrestricted modification searching–based filtering was enabled. The resulting identified PSMs with a *p* value  ≤0.01 and n_ptm zero were considered as novel peptides for further analysis. This analysis revealed 1,558 of the 4,387 novel peptides passing the screen. The novel peptides were distributed across all chromosomes and not restricted to any particular chromosomes or chromosomal locations, although some chromosomes such as chromosomes 1, 3, 12, and 17 carried a larger number (Fig. 1*B*).

We further attempted to verify the novel peptide sequences identified with PepQuery analysis through an additional validation tool—DeepMass:Prism, which is neural network–based deep learning prediction tool. Given a peptide sequence, DeepMass:Prism predicts fragment ion intensities, which can be used to compare experimental MS/MS spectral intensities of the novel peptides. However, as DeepMass:Prism is modeled on the MS/MS spectra of unmodified tryptic peptides, the *m/z* values do not carry iTRAQ and other modifications. To overcome this limitation, we first checked the model by externally adding the mass correction to the predicted *m/z* values (in pilot analysis) and then carried out only intensity correlation between the PepQuery-validated novel peptide identifications and experimental dataset of novel peptides and the corresponding predicted dataset (from DeepMass:Prism). Although we could retrieve the predicted spectral intensities for the entire novel peptide sequence input, the fragment-wise significant intensity correlation (Pearson correlation coefficient = 0.5–1.0) could be observed only with about 20% of the novel peptide sequences tested (supplemental Fig. S1). While we investigate further the applicability of DeepMass:Prism for novel peptide identifications from proteogenomic analysis, we have used only PepQuery-verified novel sequences to understand the biological significance of the novel peptides.

**Fig. 2:**
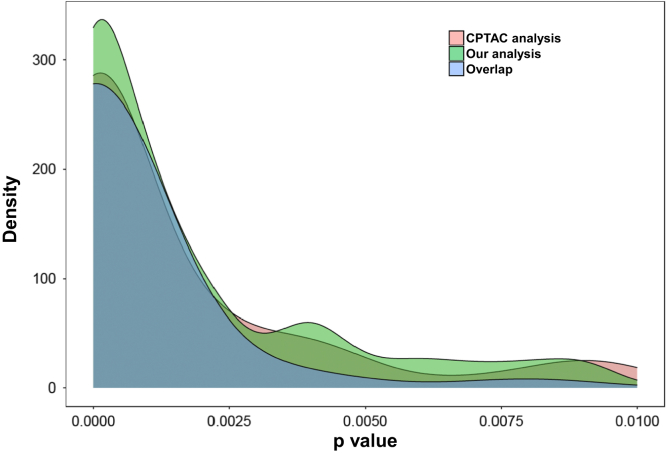
**Density distribution plot (*p* value) of novel peptides identified in proteogenomic analysis by CPTAC and those from our analysis.** Details about the number of peptides identified in our analysis as compared with CPTAC analysis are as follows: CPTAC—422, our analysis—1,558, and overlap—38. The basis and details of these numbers are given in the Results section.

We then compared our findings with that of the CPTAC study published earlier (10). In their study, Philipp *et al.* (10) generated custom database using QUILTS proteogenomic database tool. The custom database included somatic and germline single nucleotide variants, and RNA-Seq predicted junctions and fusion genes obtained based on human reference genome assembly. Spectrum Mill was used to search the proteomics data against the custom database to identify splice variants. The CPTAC study revealed 672 peptides, out of which 250 were found to be peptides identified from the known protein sequences as per RefSeq Release 96, and 422 being novel. When we compared the list of novel peptides obtained in our analysis through PepQuery validation (1,558) with 422 CPTAC novel peptides, we found 38 novel peptides to be overlapping between the two datasets, others being new identifications in our analysis. The difference in total number of novel peptides between this study and CPTAC is likely because of CPTAC dataset that included only sequences of predicted splice junctions. We then ran PepQuery with 422 novel peptides of CPTAC against corresponding raw files and obtained *p* values for the respective peptides. We examined the *p* value distribution of the PepQuery-validated dataset of novel peptides from our analysis and CPTAC analysis (overlapping and nonoverlapping) and observed the distribution to be highly correlating. Figure 2 shows *p* value density distribution of CPTAC novel peptides passing PepQuery validation (n = 151 of 422), peptides from our analysis passing PepQuery validation (n = 1,558), and peptides overlapping between our study and CPTAC (n = 38).

Thus, we report that the dataset of 1,558 novel peptides revealed through PepQuery, suitable to get biological and clinical insights. We believe extended validation analysis with the remaining peptides may provide additional information on the same line. The total list of PepQuery-validated peptides is provided in supplemental Table S2*A*, and their detailed classification is provided in supplemental Table S2, *B*–*D*) along with relevant details such as peptide sequence, the parent genes, and number of PSMs. The table includes their grouping under protein-coding and noncoding genes (*e.g.*, pseudogenes) as well as sequences yet uncharacterized for expression into proteins (potential nORFs). Various categories of novel peptides from events such as 5′ or 3′ UTR, exon extensions, exon skipping, frame shifts, intronic, and others are shown in the table. Their chromosome-wise distribution and sequence coordinates on the chromosomes are also included. The RNA-level expression values corresponding to the PepQuery-validated novel peptides are given in supplemental Table S3.

### Novel Peptides From Yet Uncharacterized (Potential ORFs) Regions

Our analysis indicated that of the total 1558 novel peptides, 831 peptides with 2269 PSMs were found to be part of sequences from outside any canonical protein-coding or known noncoding genes of the human genome. A majority of these sequences were found to be located in the intronic regions of the known protein-coding and noncoding genes. Some of them were located in the 5′ and 3′ UTR regions of known genes, whereas a few were found to be in a completely different frame within the annotated protein sequences. The biological validity of these novel peptides, their relationship with the novel short and long ORFs reported in the literature (38), or their functional implication is not clear and stands to be investigated.

### Novel Peptides From Known Genes—Protein-Coding and Noncoding Genes

**Fig. 3:**
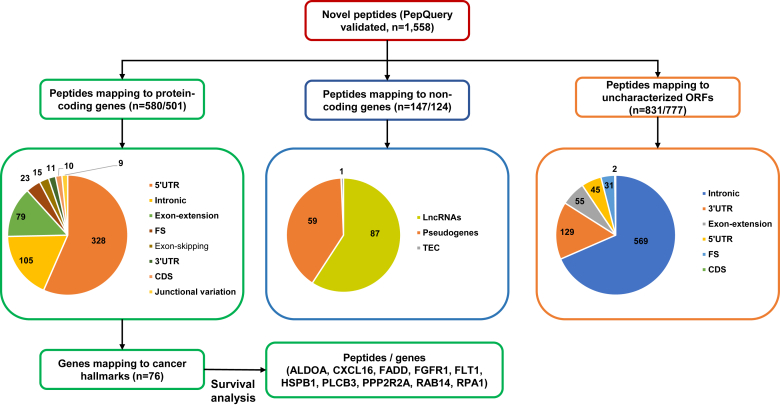
**Schematic representation of novel peptide categories to understand their functional and clinical significance.** Of the 1,558 peptides validated by PepQuery (Fig. 1), 580 were found to map to known protein-coding genes,147 mapped to noncoding genes, and 801 mapped to uncharacterized ORFs. The different types of peptides seen in each of the categories along with the respective numbers are depicted using the pie chart. The peptides (n = 580) corresponding to 501 protein-coding genes were further mapped to cancer hallmarks to identify their tumor relevance. Seventy-six of them mapped to cancer hallmarks, and the corresponding novel peptide sequences were further subjected to survival analysis as described under Experimental Procedures and Results sections. The survival association plots for significant peptide sequences are given in Figure 4.

After filtering the peptides belonging to the undefined and uncharacterized noncoding sequences (potential nORFs) from 1,558 novel peptides, we obtained 727 peptides considered to be belonging to the annotated gene sequences. We used the tool ACTG to map these novel peptides back to the annotated sequences of the human genome on ENSEMBL and found 580 being protein-coding genes and the rest 147 mapping to noncoding genes such as pseudogenes and other noncoding transcripts such as lncRNAs. As shown in Figure 3, 580 novel peptides mapping to protein-coding sequences provided evidence for alternate translation start sites, changes in the annotation of exon boundaries, and novel splice events within the genes. Importantly, they included peptides because of alternative splicing events, such as exon skipping (peptides spanning two nonconsecutive exons providing evidence for a skipped exon), exon extensions (peptides that either partly extend to the intron or are only intronic peptides), 5′UTR and 3′ UTR peptides, and junctional variations. A total of 147 peptides with 1,173 PSMs were identified mapping to 124 annotated noncoding transcripts. These included 59 peptides corresponding to 39 pseudogenes, 87 peptides corresponding to 84 lncRNAs, and a single peptide corresponding to 1 “To be experimentally confirmed transcript” (TEC).

Among the proteins covering 580 novel peptide sequences identified, we wanted to identify those that had any association with cancer-related biological processes. So, we mapped the proteins corresponding to the novel peptides to cancer hallmark gene sets (http://bio-bigdata.hrbmu.edu.cn/CHG/nav_download.html) and found 76 of them mapped to cancer hallmarks. These proteins included several key oncogenes, [Erb-B2 receptor tyrosine kinase 2, fibroblast growth factor receptor (FGFR), and DEAD box helicase 5 (DDX5)], transcription factors[cyclic adenosine monophosphate response element-binding protein (CREBBP), mediator complex subunit 1(MED1), and mediator complex subunit 16 (MED16)], protein kinases [mitogen-activated protein kinase kinase 1(MAP2K1), mitogen-activated protein kinase 13(MAPK13) and casein kinase 1 epsilon (CSNK1E)], cell adhesion/differentiation or cell surface molecules, as well as a cytokine, C-X-C motif chemokine ligand 16 (CXCL16). Aldolase A (ALDOA), phospholipase C beta 3 (PLCB3), and protein phosphatase 2 regulatory subunit B alpha (PPP2R2A) are some of the enzymes with regulatory significance. The details of these annotations are given in supplemental Table S4).

In our analysis, we could also identify expression of many noncoding sequences particularly pseudogene peptides. We observed 59 novel peptides mapping to 39 pseudogenes. We cross-verified some of these peptide sequences—CYP2B7P, IGHGP, and RHOXF1P3, for their presence in the MS/MS data of an additional BRCA proteome study of Johansson *et al.* (39). The dataset consisted of tandem mass tag–based quantitative proteomics data obtained using 45 breast tumor specimens of various subtypes. These genes were also confidently identified in this additional dataset, thus supporting the validity of their expression.

### Survival Analysis of Novel Peptides

**Fig. 4:**
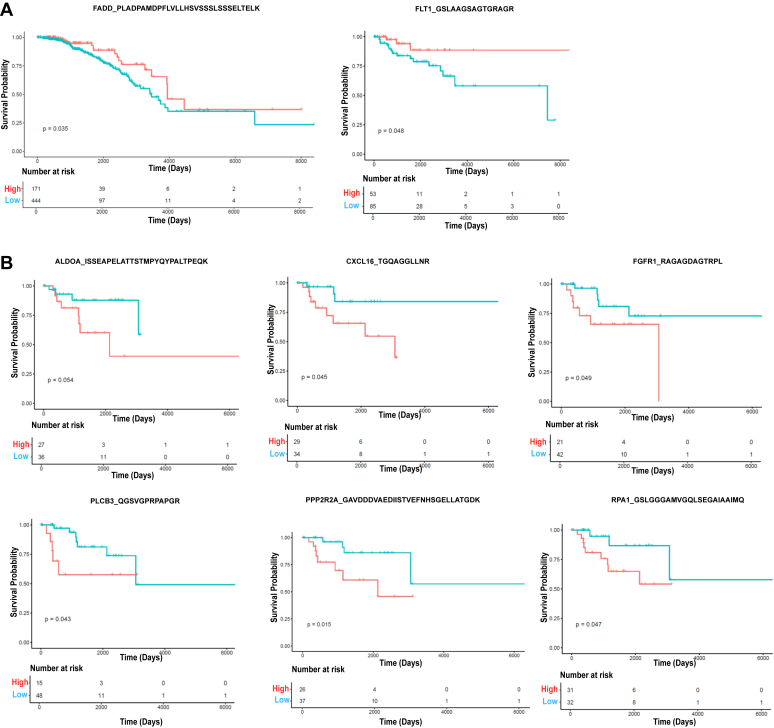
**Survival analysis of novel peptide sequences mapping to protein-coding genes.** Survival plots for the novel peptide sequences belonging to eight genes, namely FADD, FLT1, ALDOA, CXCL16, FGFR1, PLCB3, PPP2R2A, and RPA1, found to exhibit significant survival association are provided along with the respective peptide sequences. The novel peptides were quantified at transcript level using the breast cancer RNA-Seq data from TCGA. *Red line* represents high-expression group of patients, whereas *blue line* indicates low-expression group of patients. Number of patients at risk in the high- and low-expression groups are also shown. *A*, peptides showing survival association in luminal (FADD) and basal subtypes (FLT1). *B*, peptides showing survival association in HER2-enriched subtype (ALDOA, CXCL16, FGFR1, PLCB3, PPP2R2A, and RPA1). ALDOA, aldolase A; CXCL16, C-X-C motif chemokine ligand 16; FADD, Fas-associated death domain; FGFR1, fibroblast growth factor receptor 1; FLT1, Fms-related receptor tyrosine kinase 1; HER2, human epidermal growth factor receptor 2; PLCB3, phospholipase C beta 3; PPP2R2A, protein phosphatase 2 regulatory subunit B alpha; RPA1, replication protein A1.

BRCA is one of the most common cancer of the females with heterogenous pathology and broadly comprises of four subtypes, namely, luminal, basal or triple negative, HER2-enriched and normal like, which differ in the expression of hormone receptors and are prognostically different. The novel peptide sequences mapping to cancer hallmark genes were quantified at transcript level using total TCGA BRCA RNA-Seq data, as detailed under the Experimental Procedures section, and survival analysis was carried out in subtype-specific manner. On the basis of *p* value cutoff (≤0.01) for the expression of these 76 genes, 57 peptides were selected, and the patients were stratified into high- and low-expression groups based on the mean gene expression value, and survival analysis was performed. A total of 26 peptides were found to be associated with survival on the basis of significant (≤0.05) *p* values. Furthermore, manual quality evaluation of the respective MS/MS spectra of these candidates revealed 10 of them to be of acceptable quality. An independent survival analysis of the respective parent genes of these 10 candidates were also performed (supplemental Fig. S2), and we observed the parent genes of two novel peptides—HSPB1 (heat shock protein family b [small] member 1) and RAB14 (RAB14, member RAS oncogene family)—showing near-significant survival association (*p* = 0.1). Therefore, a peptide was considered to be significantly associated with survival if its *p* value cutoff was ≤0.05, it passed through a manual check for spectral quality, and if its parent gene did not show any survival association. Thus, we found eight novel peptides corresponding to eight genes to be significantly associated with survival as shown in Figure 4 and are listed in Table 2, with the details in supplemental Table S5. Of these sequences, Fms-related receptor tyrosine kinase 1 (FLT1) is associated with survival of basal subtype and Fas-associated death domain (FADD) protein with luminal type. On the other hand, we observed high expression of six novel peptide sequences (ALDOA, CXCL16, FGFR1, PLCB3, PPP2R2A, and replication protein A1 (RPA1) to be distinctly associated with poor survival of HER2-enriched subtype. This subtype is estrogen receptor and progesterone receptor negative but HER2 positive and is one of the aggressive subtypes. Interestingly, all these novel peptide sequences map to 5′UTR except PPP2R2A, and the parent genes of many of them are associated with multiple (more than four) hallmarks of cancer implicating their determinant role in tumor pathogenesis.

**Table 2:** List of novel peptides corresponding to protein-coding genes significantly associated with survival

Serial number	Novel peptide sequence	Gene symbol; gene description	Molecular function	CHG class	Survival association	Survival outcome	*p* (survival)	
							Novel peptide	Parent gene
1	ISSEAPELATTSTMPYQYPALTPEQK	ALDOA; aldolase, fructose-bisphosphate A	Lyase activity	Reprogramming energy metabolism	HER2-enriched	High expression of poor survival	0.05	0.31
2	TGQAGGLLNR	CXCL16; C-X-C motif chemokine ligand 16	Chemokine activity	Inducing angiogenesis; evading immune destruction; resisting cell death; and tumor-promoting inflammation	HER2-enriched	High expression of poor survival	0.05	0.44
3	RAGAGDAGTRPL	FGFR1; fibroblast growth factor receptor 1	Transmembrane receptor protein tyrosine kinase activity	Inducing angiogenesis; sustaining proliferative signaling; resisting cell death; activating invasion and metastasis; enabling replicative immortality; evading growth suppressors; and reprogramming energy metabolism	HER2-enriched	High expression of poor survival	0.05	0.88
4	AESASMTER^a^	HSPB1; heat shock protein family b (small) member 1	Chaperone activity	Resisting cell death; activating invasion and metastasis; inducing angiogenesis; sustaining proliferative signaling	HER2-enriched	High expression of poor survival	0.03	0.12
5	QGSVGPRPAPGR	PLCB3; phospholipase C beta 3	Phospholipase activity	Enabling replicative immortality; activating invasion and metastasis; resisting cell death; sustaining proliferative signaling; tumor-promoting inflammation; reprogramming energy metabolism; and evading immune destruction	HER2-enriched	High expression of poor survival	0.04	0.35
6	GAVDDDVAEDIISTVEFNHSGELLATGDK	PPP2R2A; protein phosphatase 2 regulatory subunit B alpha	Protein serine/threonine phosphatase activity	Activating invasion and metastasis; evading growth suppressors; reprogramming energy metabolism; sustaining proliferative signaling; and resisting cell death	HER2-enriched	High expression of poor survival	0.02	0.40
7	PSHQQPPSATMATAPYNYSYIFK^a^	RAB14; Ras-related protein Rab-14	GTPase-binding activity	Reprogramming energy metabolism	HER2-enriched	High expression of poor survival	0.01	0.08
8	GSLGGGAMVGQLSEGAIAAIMQ	RPA1; replication protein A1	DNA binding	Genome instability and mutation	HER2-enriched	High expression of poor survival	0.05	0.41
9	PLADPAMDPFLVLLHSVSSSLSSSELTELK	FADD; Fas-associated death domain	Receptor signaling complex scaffold activity	Evading immune destruction; resisting cell death; and tumor-promoting inflammation	Luminal	Low expression of poor survival	0.04	0.20
10	GSLAAGSAGTGRAGR	FLT1; Fms-related receptor tyrosine kinase 1	Transmembrane receptor protein tyrosine kinase activity	Inducing angiogenesis; activating invasion and metastasis; sustaining proliferative signaling; evading growth suppressors; and resisting cell death	Basal	Low expression of poor survival	0.05	0.21

a Parent genes, HSPB1 and RAB14, were found to have near significant survival association. Please refer to supplemental Fig. S2.

**Fig. 5:**
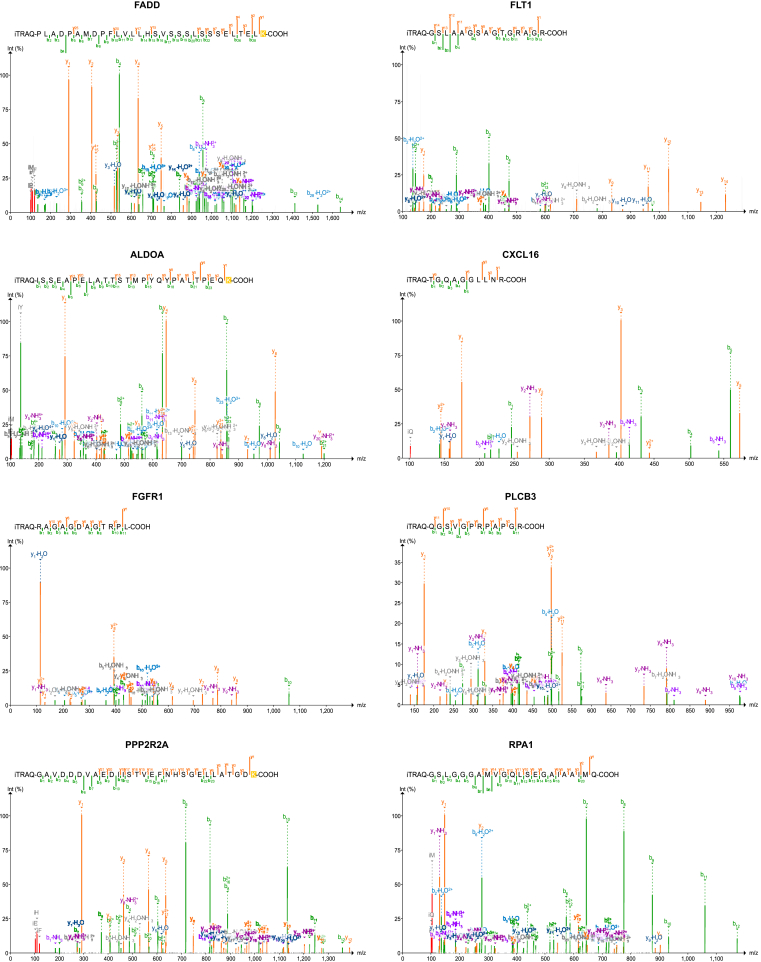
**MS/MS spectra of novel peptides of eight protein-coding genes with survival association as shown in**Figure 4**.** The details of the peptides and their corresponding genes are given in supplemental Table S5.

The sequence and the MS/MS spectra of eight survival-associated novel peptides are shown in Figure 5.

## Discussion

The CPTAC has recently carried out proteogenomic analysis of BRCA multiomics data (10). They have drawn genomics and transcriptomics data from TCGA resource for 105 specimens of BRCA of different subtypes and generated proteomics data for the same samples using high-resolution MS. The transcriptomics and proteomics data were integrated to identify novel peptides originating from transcript sequences spanning junctional sequences representing novel splice events. We have used the same transcriptomics and proteomics data, available at the CPTAC resource and implemented a proteogenomic pipeline developed by us, for genome-wide identification of novel peptides that represented alternative splice events as well as translation of other sequences in the genome. While we present the landscape of these novel peptides from coding and noncoding sequences, their types, chromosome-wise distribution, we have also analyzed a selected subset to get insights on their potential functional and clinical relevance.

Several of the novel peptide sequences belong to the known protein-coding genes, which included several oncogenes, transcription factors, kinases, cell invasion molecules, and others, as well as have roles in hallmarks of cancer, (supplemental Tables S2*B* and S5). Many novel peptides identified from the noncoding genes belong to sequences that corresponded to the pseudogenes and lncRNAs (supplemental Table S2*C*). The functional significance of the expression of these pseudogenes is not clear, but the observation permits some discussion. Expression from pseudogenes is known to occur under certain normal or pathological conditions including cancer. An integrative meta-analysis of the transcription data for many cancer types revealed expression for about 2,000 or more distinct pseudogenes (7). Any functional relevance of the expression of pseudogenes is a matter of debate. Increasing evidence from the recent literature suggests the potential roles of pseudogenes in regulating cognate wildtype gene expression/function by multiple ways—generation of endogenous siRNAs, antisense RNAs, or sequestering miRNA, competitive inhibition of translation of wildtype transcripts, and there may be other ways. Thus, pseudogene expression merits deeper exploration of their possible roles in biology and in human disease.

An even larger number of novel peptides mapped to the novel and yet uncharacterized sequences of the human genome (supplemental Table S2*D*). During past decade, the noncoding part of the human genome has been shown to harbor information for novel genes such as lncRNAs, and even protein expression from these yet uncharacterized sequences has been reported in the literature (38). Their annotation is challenging because of the present conventions for defining an ORF in terms of their expected length. Many of these sequences are now shown to have start and stop codons, and the expression products are also known to form protein-like structures and even undergo post-translational modifications. Much of the experimental support for the existence of such expressions comes from Ribo-Seq data or MS and thus qualifying these sequences to be regarded as potential nORFs. sORFs are short up to 100 amino acids in length, and alternate ORFs roll out proteins in alternate frames to known proteins. The number of these expressions is growing such as to revisit the question of the coding potential of the noncoding DNA so believed until now and the conventional definitions of an ORF. The biological significance of these nORFs is not fully understood. Studies of the TCGA and Genotype-Tissue Expression consortiums have helped identification of large number of transcripts containing nORFs from several cancer types and are also reported to be dysregulated in cancer tissue. Many of them functionally associated with the hallmarks of cancer can act as oncogenic factor promoting cell proliferation or as tumor suppressors, and their differential expression may be associated with disease prognosis. While the identification of the novel peptides mapping to intronic regions or 5′ or 3′ untranslated regions of such sequence regions need rigorous analytical verification, they deserve detailed characterization in terms of the sequence composition, the chromosomal and gene context, their dysregulation, and impact on BRCA and its subtypes.

We analyzed the novel peptides mapping to protein-coding genes and cancer hallmarks for survival analysis based on their patient-wise RNA expression profile. After carefully evaluating the initial results as detailed under the Results section, we have shortlisted eight novel peptide sequences to be significantly associated with patient survival. The novel peptides identified, the corresponding parent genes/proteins, their key molecular functions, and observed survival association have been listed in Table 2.

High expression of FLT1 is associated with poor prognosis of basal subtype of BRCA, whereas that of FAAD with luminal subtype. FLT1 belongs to the vascular endothelial growth factor receptor family of tyrosine kinase receptors and is involved in the regulation of angiogenesis (40). Overexpression of FLT1 and its two ligands, placenta growth factor and vascular endothelial growth factor receptor B, has been reported in different tumors including BRCA and shown to be associated with poor prognosis (40, 41). FADD is an ubiquitous protein encoded on chromosome 11. With its death effector protein interaction domains, it facilitates interactions between Fas receptor and members of the caspase cascade inducing apoptotic signaling. During the last decade, FADD has been shown to have a role in many signaling complexes and thus is implicated in other processes, such as proliferation, cell cycle regulation, and development as well as in gene expression changes and regulation of metabolic processes. Changes in the expression of FADD and its post-translational modifications seem to occur in many cancers (42), but how it functions in cancer is complex as both overexpression and downregulation of FADD in cancers has reported BRCA to being one of the high-expressing cancer. Chromosomal amplification is associated with BRCA and is the most likely factor for higher expression. Anticancer drugs, tamoxifen and paclitaxel, in BRCA have been reported to activate FADD phosphorylation resulting in cell cycle arrest and suppression of cancer growth through p53 stabilization. Overall, it is clear that considering the multifunctional roles of FADD and its dynamics in cancer, it qualifies as a strong candidate marker for diagnosis, prognosis, or therapeutic strategies.

Distinct among these novel peptides is a panel of six peptide sequences showing survival association with HER2-enriched subtype of BRCA, one of the more aggressive types. The parent genes corresponding to these novel peptide sequences include several regulatory enzymes (ALDOA, PLCB3, and PPP2R2A), a growth factor receptor kinase (FGFR1), a DNA-binding factor (RPA1), as well as a cytokine (CXCL16) involved in immune modulation. Aldolase is one of the glycolytic enzymes with three isoenzymes (A, B, and C) that are developmentally regulated (43). ALDOA being the adult form. Recently, a new role of ALDOA in cancers has been proposed through its association with genes relevant to cell cycle independent of glycolysis and thus with the development and prognosis of several cancers. Other than its function in glycolysis and energy generation, ALDOA also contributes to other functions, such as regulation of cell shape and motility, actin cytoskeleton organization, and regulation of cell proliferation. Overexpression of ALDOA is believed to enhance glycolysis in tumor cells, promoting their growth. In laryngeal squamous cell carcinomo, its upregulation correlates with metastasis and poor prognosis, whereas its downregulation reduces tumor cell motility and tumorigenesis. PPP2R2A (protein phosphatase 2A) belongs to the family of serine/threonine phosphatases. It is a heterotrimeric protein consisting of a highly conserved catalytic domain that binds to different regulatory subunits to exert its functions. PPP2R2A codes for an alpha isoform of the regulatory subunit B55 subfamily and is known to be involved in regulation of cell cycle (44). Deregulation of PPP2R2A has been implicated in many cancers (45). PLCB3 is a member of highly conserved phospholipase family. This enzyme catalyzes the hydrolysis of phosphatidylinositol-4,5-bisphosphate (PIP_2_) to produce the second messengers, inositol-1,4,5-triphosphate and diacylglycerol in G protein–coupled receptor–mediated signal transduction (46). High expression of *PLCB3* mRNA has been shown to be associated with poor survival in non–small cell lung cancer patients (47). Fibroblast growth factors (FGFs) are broad-spectrum mitogens and regulate a wide range of cellular functions, including migration, proliferation, differentiation, and survival. FGFs and FGFRs have been identified in the cancer vasculature and supporting stromal cells as well as cancer cells (48) The FGFR family consists of tyrosine kinase receptors involved in several biological functions, such as differentiation, proliferation, and apoptosis of various types of cells. Alterations of FGFR have been reported to be important for progression and development of several cancers and as attractive targets. In addition to post-translational modifications, alternative splicing and translational initiation generate multiple isoforms of FGFs/FGFRs and regulate their expression levels and binding specificity to individual FGFs. RPA1 is a highly conserved heterotrimeric single-stranded DNA-binding protein complex that plays a significant role in maintaining the genome integrity by facilitating DNA replication, recombination, and repair (49). Chen *et al.* (50) modulates the function of DNA helicases, fork remodeling, checkpoint activation, and telomere maintenance (51). The RPA1 complex is composed of three subunits—RPA1, RPA2, and RPA3 with six DNA-binding domains, involved in DNA protein and protein–protein interactions during DNA damage and repair processes. The crosstalk between immune system and cancer cells in the tumor microenvironment is mediated by cytokines and chemokines during cancer initiation and progression (52). CXCL16 is a small cytokine belonging to the CXC chemokine family and has been identified to bind with the C-X-C chemokine receptor type 6. CXCL16 is produced by macrophages and dendritic cells expressed as not only a membrane-bound molecule but also a soluble chemokine. The membrane-bound CXCL16 is released after proteolytic cleavage, and stimulation of C-X-C chemokine receptor type 6 with soluble CXCL16 facilitates cytoskeletal rearrangement during BRCA cell migration and invasion and regulates immune cell chemotaxis into CXCL16-enriched environments.

Unlike in the case of the novel peptides mapping to heat shock protein family b (small) member 11 and RAB14 (see Results section), the parent genes of the novel peptides mapping to the above proteins did not show survival association (supplemental Fig. S2), permitting the conclusion that the survival association of the novel peptides observed and discussed previously is likely because of the novel peptide sequence itself. What is the effect of the novel peptide sequence on the expression or function of the gene/protein and how they contribute to the isoform spectrum of the relevant protein are not clear. However, it is interesting to note that the novel peptides of the genes, ALDOA, CXCL16, FGFR1, PLCB3, and RPA1, associated with HER2-enriched subtype, mapped to the 5′UTR region indicating 5’ (N-terminal region) extension of these proteins resulting from an in-frame upstream translation initiation site. (The novel peptide corresponding to PPP2R2A provided evidence for deletion of an alanine at the junction of exon 2 and exon 3.). The 5′ and 3′UTRs of eukaryotic genes have regulatory role in the process of translation. Sequences at the 5′ UTR particularly are involved in binding and selection of the start site for translation and formation of the initiation complex through interaction with the initiation factors and 3′UTR (which is also involved in regulating the stability of the mRNA). Interestingly, in all these cases, the extended N-terminal sequence lacked an upstream methionine, which is generally a degradation signal in cells. Thus, a question that could be asked is whether 5′ extensions observed in the five of these survival-associated sequences influence the molecular interactions to bring about the change in the translational efficiency of the mRNA or contribute to its stability and therefore their altered levels. Similarly, the loss of alanine at the junction of exon 2 and 3 in PPP2R2A may have some structural consequence on the protein associated with malignant status. We do not have answers to these questions, but the possibilities cannot be ruled out. Nevertheless, the respective novel peptide sequences show statistically significant association with patient survival as against sequences of the parent genes, which are associated with multiple cancer hallmarks, strongly suggests their implication in breast cancer pathogenesis. Identification and characterization of the full-length proteoforms of these gene products harboring the novel peptide sequences, their expression levels using MS or antibody-based approaches, the structural and functional consequences of these novel sequences on the protein would therefore be important to investigate in a targeted manner.

## Conclusions

We developed a proteogenomics pipeline that uses custom database based on *de novo*–assembled transcripts from RNA-Seq data and their *in silico* translation in six frames. We applied our pipeline using the TCGA-BRCA RNA-Seq and the CPTAC proteome data for the same cohort of BRCA patients. Our analysis identified novel peptides representing alternative splicing of many known protein-coding genes as well as expressions of pseudogenes, lncRNAs, N-terminal extensions, and short potentially nORFs from intronic regions. These changes regarding novel expressions at the translational level may influence the function of the cognate proteins either by changes in their levels or structure-driven functional change. The translation of sequences into novel peptides may be driven by the tumorigenic condition as revealed by their survival analysis and association with favorable or unfavorable prognosis. Further targeted analysis of these novel peptides would give useful insights for their clinical application.

## Data Availability

The proteogenomics pipeline is made available as a GitHub repository (https://github.com/MSCTR/Denovo-Proteogenomics-Pipeline). The CPTAC raw data are available at https://cptac-data-portal.georgetown.edu/study-summary/S029.

## Supplemental data

This article contains supplemental data.

## Conflict of interest

The authors declare no competing interests.

## Supplementary Data

Supplemental Figure S2

Supplemental Figure S1 with Legend and Analysis
