# Supplementary figures and images for: Proteogenomic Analysis of Breast Cancer Transcriptomic and Proteomic Data, Using De Novo Transcript Assembly: Genome-Wide Identification of Novel Peptides and Clinical Implications
Source: Mol Cell Proteomics. 2022 Feb 26;21(4):100220. doi: 10.1016/j.mcpro.2022.100220 (PMC9020135; doi:10.1016/j.mcpro.2022.100220)

Supplementary Figure 1

A

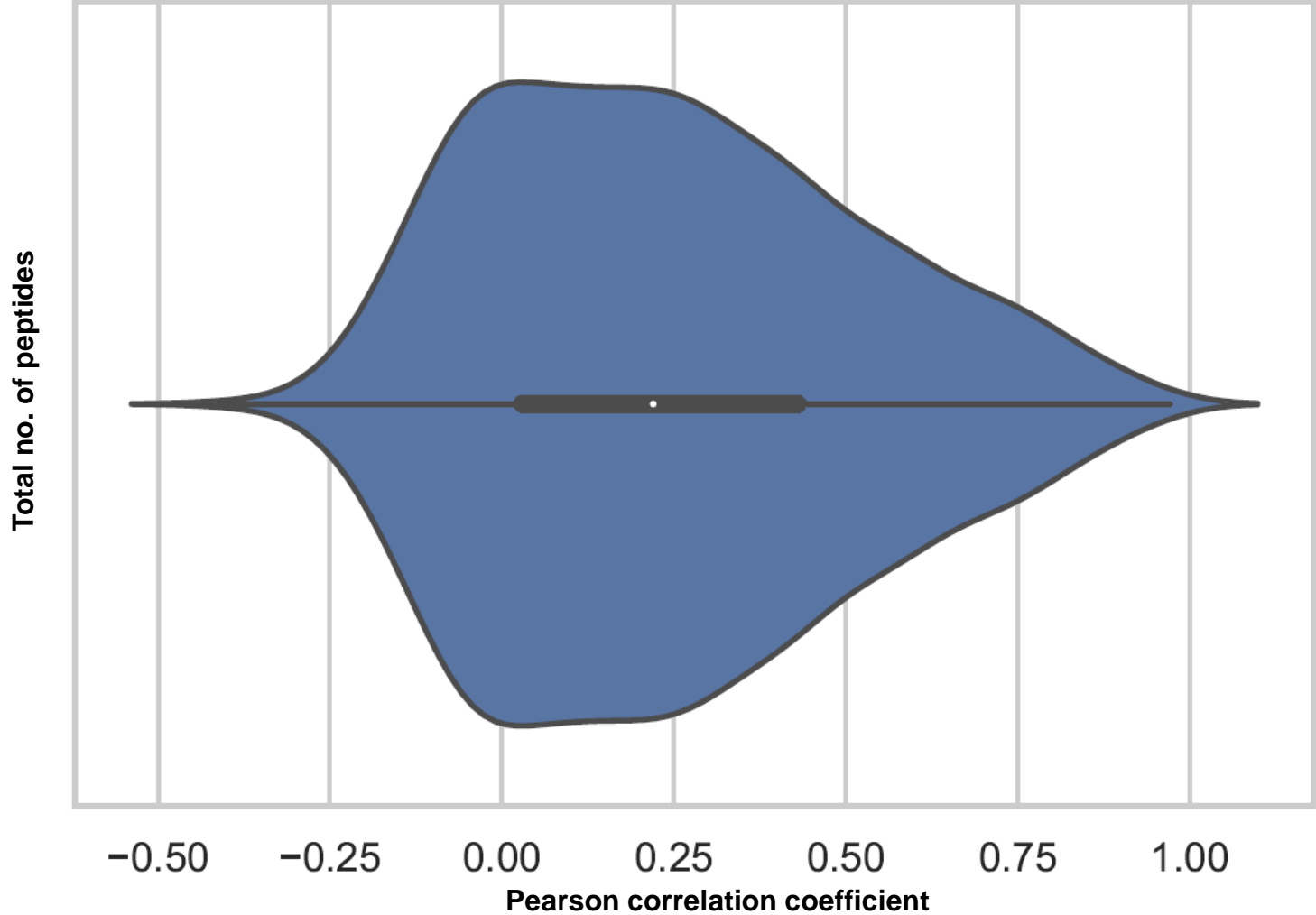

**B****VGASETEENGSDSFMHSM DPQLER (Corr:0.97)**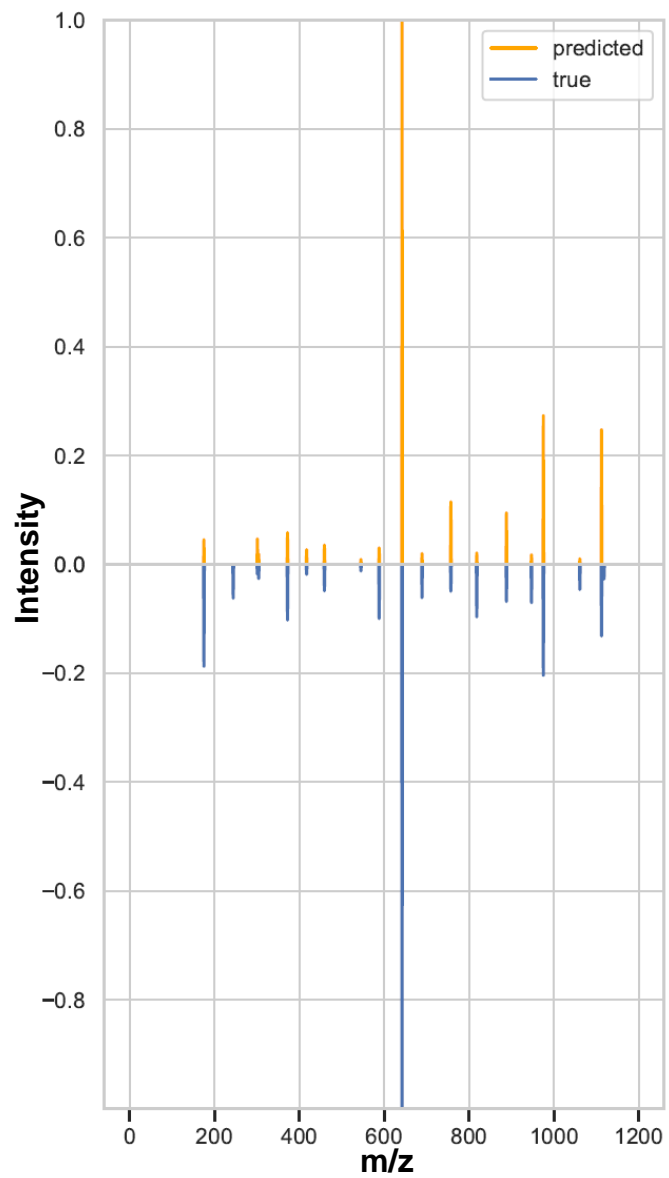**IQVGVTQVQR (Corr:0.57)**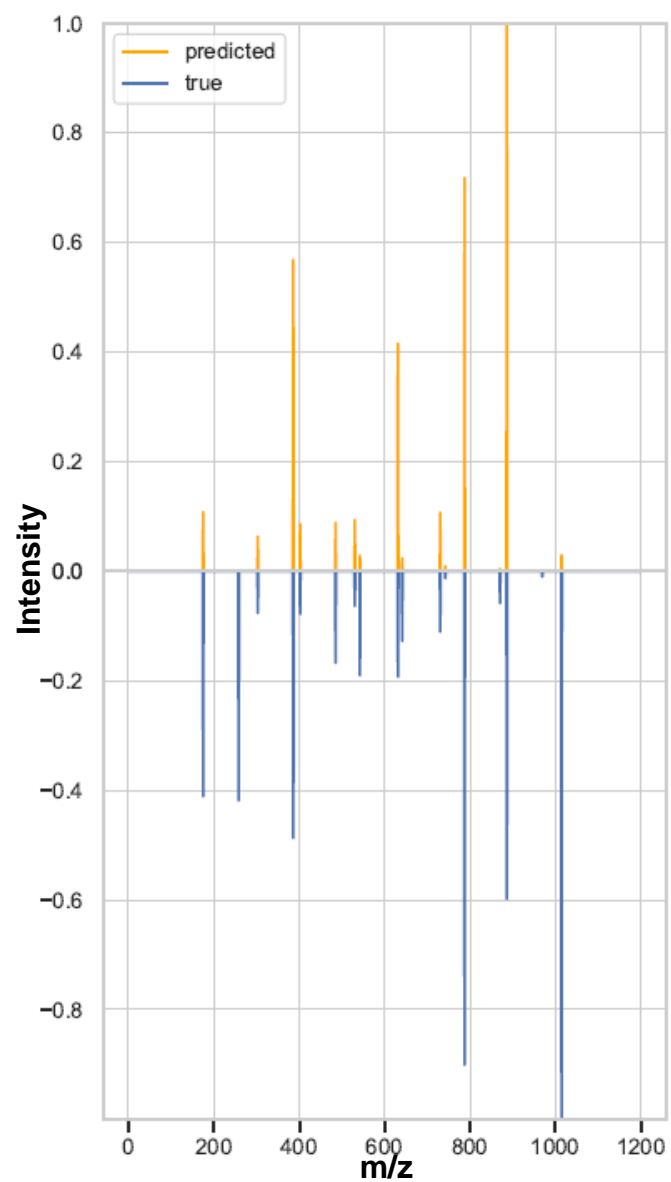**ASEEAVF (Corr:-0.41)**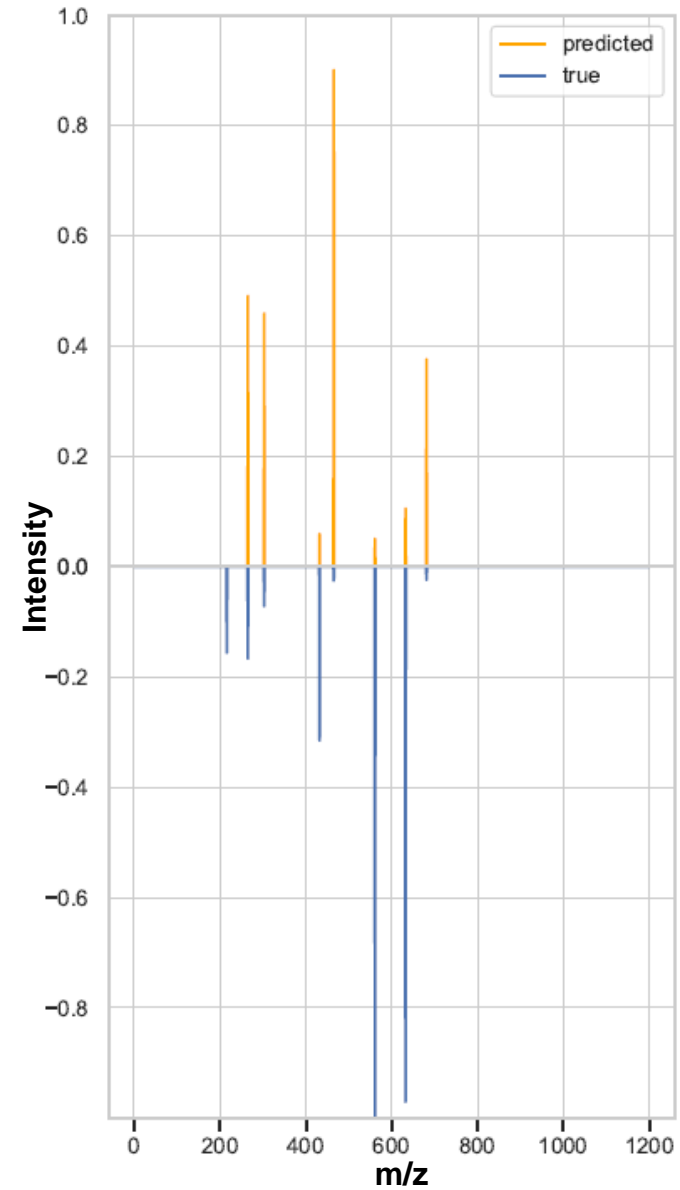

Supplement: Supplemental Figure S1 [file mmc6.pdf]

Supplementary Figure 2

A

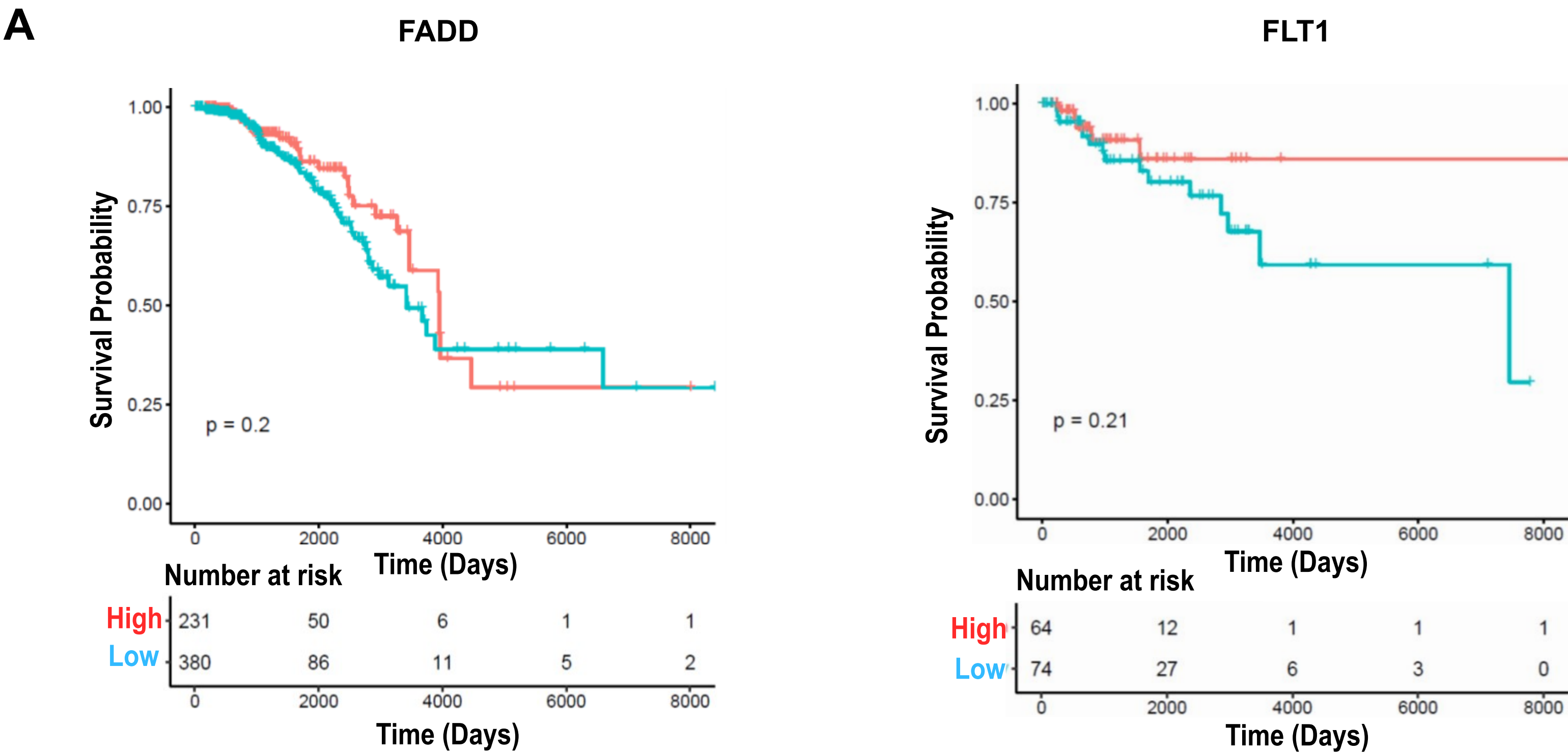

B

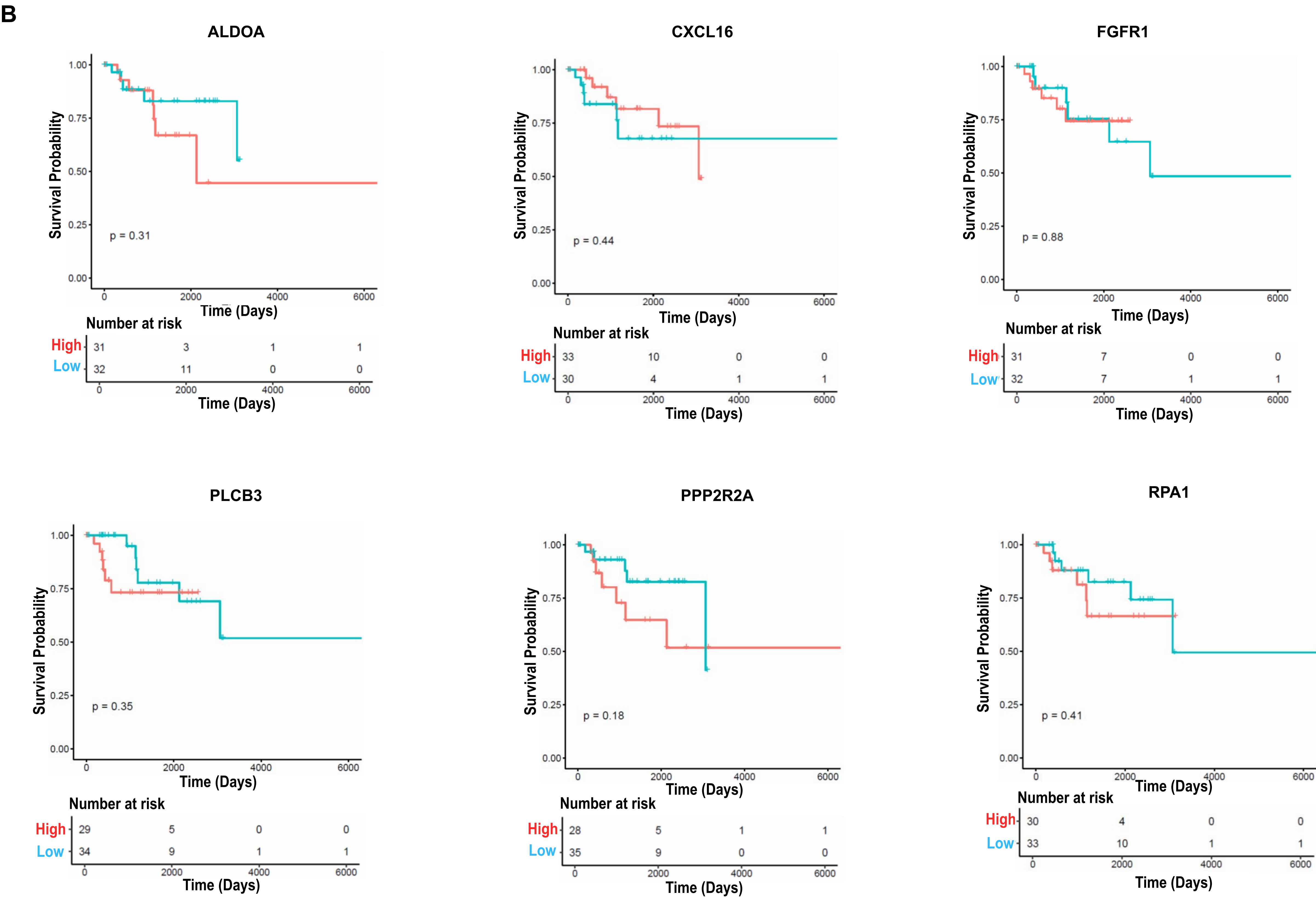

C

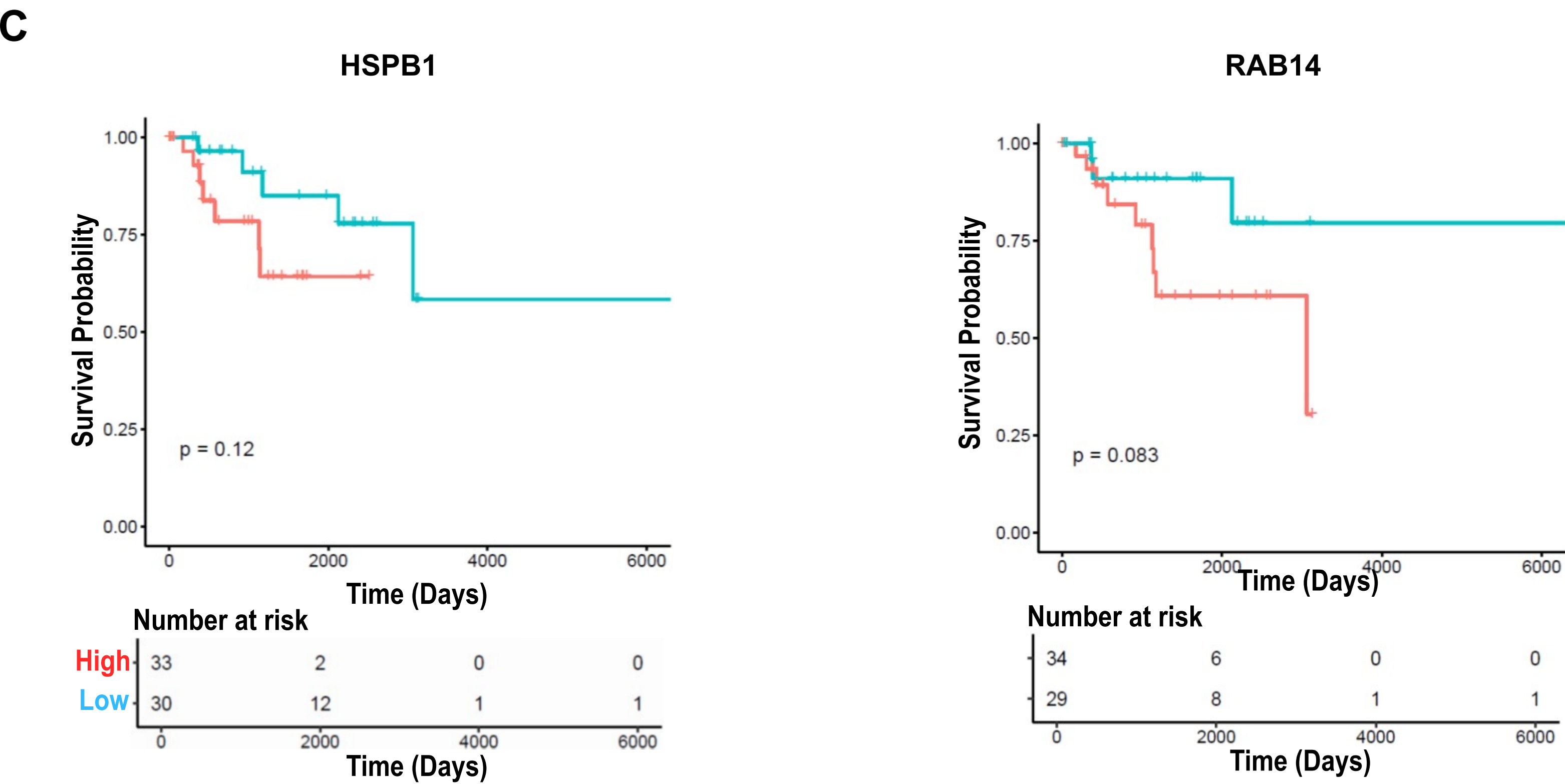

Supplement: Supplemental Figure S2 [file mmc7.pdf]
