# Supplementary material for: Proteogenomic Analysis of Breast Cancer Transcriptomic and Proteomic Data, Using De Novo Transcript Assembly: Genome-Wide Identification of Novel Peptides and Clinical Implications
Source: Mol Cell Proteomics. 2022 Feb 26;21(4):100220. doi: 10.1016/j.mcpro.2022.100220 (PMC9020135; doi:10.1016/j.mcpro.2022.100220)
Supplement: Supplemental Figure S1 with Legend and Analysis [file mmc8.docx]

**Supplementary Figure 1A**. Implementation of DeepMass:Prism prediction tool for MS/MS fragmentation spectra prediction to validate novel peptide sequences. The figure shows Pearson correlation Coefficient (PCC) distribution plot for the predicted and experimental fragment ion intensities


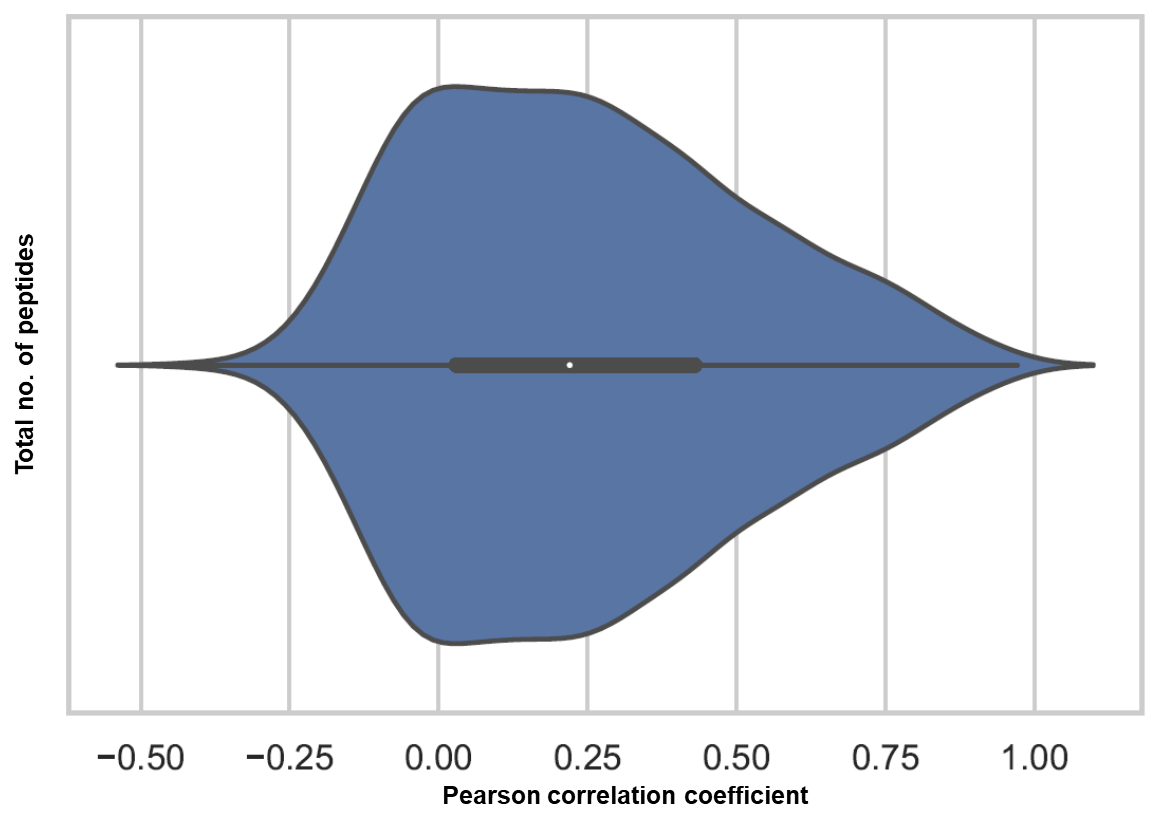


**Supplemental Figure 1B**. Mirror plots showing best, moderate and the poor correlated true and predicted fragment intensities. X axis shows m/z value ranging from 0 to 1200 and Y axis shows experimental or true intensity (indicated in blue) as per PepQuery output whereas predicted intensity (indicated in orange) are from DeepMass:Prism. Y axis shows intensity value normalized to maximum intensity between y and b ions intensity peaks ranging from 0 to 1. The spectra shown here is of y and b ions. Neutral losses as well as higher fragment ions charges are not included.


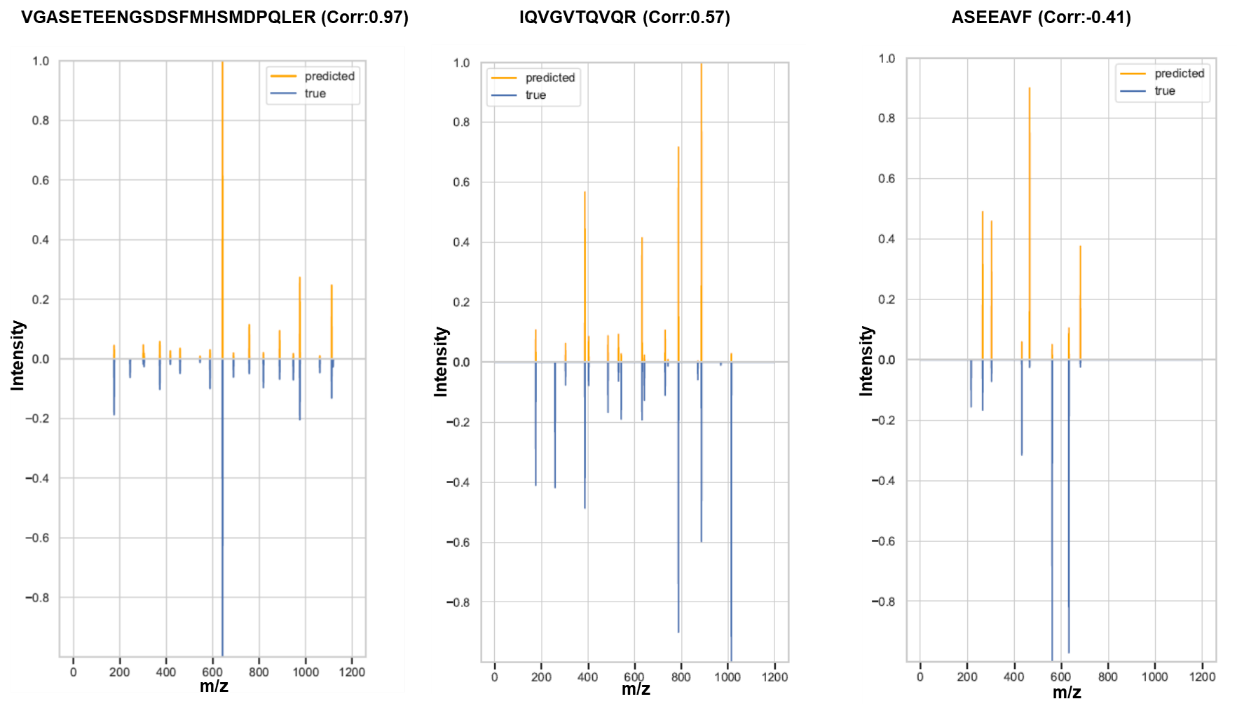


**Analysis and Observation**: In 1A, PepQuery validated novel peptide sequences (n=1,356 derived from Supplemental Table 2A) were used and run through DeepMass:Prism analysis. DeepMass:Prism was trained on unmodified tryptic peptides. However, the novel peptide dataset includes modifications due to iTRAQ labels used in the quantitation experiments. So, in the pilot analysis, we manually checked for m/z matches between the experimental MS/MS spectra and DeepMass: Prism derived fragmentation spectra of a number of novel peptides with iTRAQ mass label correction at the N terminal and lysine free NH2 groups and found complete concordance between the two. Subsequently, the b and y ion intensities were predicted from DeepMass:Prism ignoring iTRAQ label mass changes. DeepMass:Prism prediction output contains y and b ions with fragment ion charge 1. So, for correlation coefficient calculation we considered only y and b ions with fragment ion charge 1 and ignored fragment ions with neutral losses and ions with charges more than 1. The predicted intensities were then compared to the intensities provided by PepQuery output. We calculated correlation coefficient for each peptide, using intensities provided by PepQuery output and predicted intensities from DeepMass:Prism. The median PCC is 0.22 as shown in the figure.

In **1A**, surprisingly only about 20 % peptides (n=233) showed PCC greater than 0.5 although these sequences had undergone validation through PepQuery. Rest of the peptides have either very low PCC value or are negatively correlated. In **1B,** the representative mirror plots show high, moderate and poor correlated spectra. In the plot with poor correlation (PCC= -0.41), we can see mismatch between the intensity peak in both experimental as well as predicted spectrum.

The images of the experimental MS/MS spectrum matches of the novel peptides with survival association shown in Figure 5 of the main text support their good quality in terms of number of annotated ions, their S/N ratio and the sequence contiguity. However, only one of them (ALDOA) had good PCC value of 0.7, 4 of them, PPP2R2A, PLCB3, RPA1 and CXCL16 had low PCC of 0.33, 0,37, 0.02 and 0.28 respectively, in the above correlation analysis (not shown here) and 3 were not predicted by DeepMass:Prism. Such discrepancies may arise either due to a limitation in the prediction model or that in the experimental output. It is to be noted that DeepMass:Prism is modelled on unmodified spectra and may need further investigation to make it applicable to MS/MS datasets with iTRAQ modifications.
