# Supplementary material for: Proteogenomic Analysis of Breast Cancer Transcriptomic and Proteomic Data, Using De Novo Transcript Assembly: Genome-Wide Identification of Novel Peptides and Clinical Implications
Source: Mol Cell Proteomics. 2022 Feb 26;21(4):100220. doi: 10.1016/j.mcpro.2022.100220 (PMC9020135; doi:10.1016/j.mcpro.2022.100220)
Supplement: Supplemental Figures S1 and S2, Supplemental Tables S1–S5 [file mmc9.docx]

**Supplemental Figures**

**Supplemental Figure 1.**

**A.** **Implementation of DeepMass:Prism prediction tool for MS/MS fragmentation spectra prediction to validate novel peptide sequences.** The figure shows Pearson correlation Coefficient (PCC) distribution plot for the predicted and experimental fragment ion intensities

**B. Mirror plots showing best, moderate and the poor correlated true and predicted fragment intensities.** X axis shows m/z value ranging from 0 to 1200 and Y axis shows experimental or true intensity (indicated in blue) as per PepQuery output whereas predicted intensity (indicated in orange) are from DeepMass:Prism. Y axis shows intensity value normalized to maximum intensity between y and b ions intensity peaks ranging from 0 to 1. The spectra shown here is of y and b ions. Neutral losses as well as higher fragment ions charges are not included.

**Analysis and Observation**: In 1A, PepQuery validated novel peptide sequences (n=1,356 derived from Supplemental Table 2A) were used and run through DeepMass:Prism analysis. DeepMass:Prism was trained on unmodified tryptic peptides. However, the novel peptide dataset includes modifications due to iTRAQ labels used in the quantitation experiments. So, in the pilot analysis, we manually checked for m/z matches between the experimental MS/MS spectra and DeepMass: Prism derived fragmentation spectra of a number of novel peptides with iTRAQ mass label correction at the N terminal and lysine free NH2 groups and found complete concordance between the two. Subsequently, the b and y ion intensities were predicted from DeepMass:Prism ignoring iTRAQ label mass changes. DeepMass:Prism prediction output contains y and b ions with fragment ion charge 1. So, for correlation coefficient calculation we considered only y and b ions with fragment ion charge 1 and ignored fragment ions with neutral losses and ions with charges more than 1. The predicted intensities were then compared to the intensities provided by PepQuery output. We calculated correlation coefficient for each peptide, using intensities provided by PepQuery output and predicted intensities from DeepMass:Prism. The median PCC is 0.22 as shown in the figure.

In **1A**, surprisingly only about 20 % peptides (n=233) showed PCC greater than 0.5 although these sequences had undergone validation through PepQuery. Rest of the peptides have either very low PCC value or are negatively correlated. In **1B,** the representative mirror plots show high, moderate and poor correlated spectra. In the plot with poor correlation (PCC=-0.41), we can see mismatch between the intensity peak in both experimental as well as predicted spectrum.

The images of the experimental MS/MS spectrum matches of the novel peptides with survival association shown in Figure 5 of the main text support their good quality in terms of number of annotated ions, their S/N ratio and the sequence contiguity. However, only one of them (ALDOA) had good PCC value of 0.7, 4 of them, PPP2R2A, PLCB3, RPA1 and CXCL16 had low PCC of 0.33, 0,37, 0.02 and 0.28 respectively, in the above correlation analysis (not shown here) and 3 were not predicted by DeepMass:Prism. Such discrepancies may arise either due to a limitation in the prediction model or that in the experimental output. It is to be noted that DeepMass:Prism is modelled on unmodified spectra and may need further investigation to make it applicable to MS/MS datasets with iTRAQ modifications.

**Supplemental Figure 2. Survival analysis of parent protein coding genes of novel peptides with significant survival association shown in Main Figure 4.** Survival plots for the 10 protein coding genes, FADD, FLT1, ALDOA, CXCL16, FGFR1, HSPB1, PLCB3, PPP2R2A, RAB14 and RPA1 are provided. The genes were quantified at transcript level using the breast cancer RNA-Seq data in TCGA. Red line represents high expression group of patients whereas blue line indicates low expression group of patients. Number of patients at risk in the high and low expression groups are also provided. Panel A. Genes showing survival association in in luminal (FADD) and basal (FLT1) subtypes. Panel B. Genes showing survival association in Her2-enriched subtype. Panel C. Survival association plots of parent genes of novel peptides mapping to HSPB1 and RAB14 showing near significant survival association. (For details, please refer to Main Table 2).

**Supplemental Tables**

**Supplemental Table 1**

List of novel peptides (n=4,387) identified with the proteogenomics pipeline along with the respective peptide sequences, their Ensembl gene ID, gene symbol, novel peptide category, chromosomal location, variable and fixed modifications, total number of PSMs, PeptideShaker confidence score, retention time, precursor m/z, charge, theoretical mass, modified peptide sequence and spectrum ID/TCGA file name. Peptides mapping to known genes and those belonging to uncharacterized ORFs are also indicated (See Results).

**Supplemental Table 2**

**Supplemental Table 2A**

List of all peptides validated by PepQuery along with the respective peptide sequences, modifications, spectrum title, charge, experimental and theoretical mass, ppm, precursor m/z, charge, PepQuery score, p value, rank, n ptm and TCGA file name from which it was identified.

**Supplemental Table 2B**

List of all peptides validated by PepQuery mapping to protein-coding genes along with their respective peptide sequences, their Ensembl gene ID, gene symbol, gene description, peptide category, chromosomal location and total no. of PSMs.

**Supplemental Table 2C**

List of all peptides validated by PepQuery mapping to non-coding genes along with their respective peptide sequences, their Ensembl gene ID, gene symbol, gene description, gene type, peptide category, chromosomal location and total no. of PSMs.

**Supplemental Table 2D**

List of all peptides validated by PepQuery mapping to uncharacterized ORFs along with their respective peptide sequences, their Ensembl gene ID, gene symbol, gene description, gene type, peptide category, chromosomal location and total no. of PSMs.

**Supplemental Table 3**

List of novel peptides validated by PepQuery (Supplemental Table 2A) along with the median RNA expression values for normal and tumor samples obtained from TCGA, their respective log2 fold change, fold change and p value. In addition, the total number of normal and tumor samples from which the peptides are detected are provided in percentage.

**Supplemental Table 4**

List of protein coding genes corresponding to novel peptides that mapped to cancer hallmarks along with the peptide sequence, gene symbol, CHG class, peptide category, total no. of PSMs, chromosomal location and RNA expression (p value as per Supplemental Table 3)

**Supplemental Table 5**

List of novel peptides corresponding to the 10 protein-coding genes significantly associated with survival along with their peptide sequence, gene symbol, gene description, CHG class, peptide category, total no. of PSMs, chromosomal location, RNA expression (p value as per Supplemental Table 3), survival association, survival outcome, p value (survival) and parent gene survival association (p value).
